# Modifiable risk factors predicting major depressive disorder at four year follow-up: a decision tree approach

**DOI:** 10.1186/1471-244X-9-75

**Published:** 2009-11-22

**Authors:** Philip J Batterham, Helen Christensen, Andrew J Mackinnon

**Affiliations:** 1 Centre for Mental Health Research, The Australian National University, Canberra, Australia; 2 Orygen Research Centre, The University of Melbourne, Melbourne, Australia

## Abstract

**Background:**

Relative to physical health conditions such as cardiovascular disease, little is known about risk factors that predict the prevalence of depression. The present study investigates the expected effects of a reduction of these risks over time, using the decision tree method favoured in assessing cardiovascular disease risk.

**Methods:**

The PATH through Life cohort was used for the study, comprising 2,105 20-24 year olds, 2,323 40-44 year olds and 2,177 60-64 year olds sampled from the community in the Canberra region, Australia. A decision tree methodology was used to predict the presence of major depressive disorder after four years of follow-up. The decision tree was compared with a logistic regression analysis using ROC curves.

**Results:**

The decision tree was found to distinguish and delineate a wide range of risk profiles. Previous depressive symptoms were most highly predictive of depression after four years, however, modifiable risk factors such as substance use and employment status played significant roles in assessing the risk of depression. The decision tree was found to have better sensitivity and specificity than a logistic regression using identical predictors.

**Conclusion:**

The decision tree method was useful in assessing the risk of major depressive disorder over four years. Application of the model to the development of a predictive tool for tailored interventions is discussed.

## Background

Depression is a leading cause of disease burden worldwide [1,2], and is the leading risk factor for completed suicide. It frequently leads to substance abuse and lowered work productivity and is a risk factor for physical illnesses such as cardiovascular disease [3]. Despite the disease burden associated with depression, and its high personal and financial costs, knowledge about prevention lags the evidence base for treatment. Little is known about risk factors which predict the incidence, recurrence and chronicity of depression. Risk factor research has focused on specific subgroups such as the elderly or adolescents, or has been restricted to general practice (i.e., treated or help seeking) samples [4,5]. The analysis of the expected effects of a reduction of these risks over time is rarely investigated, although a few papers which break this rule using longitudinal data have begun recently to model risk reduction [4,5].

Compare this situation with what is known about the prevention of cardiovascular disease (CVD). In the CVD area, there is considerable research aimed at predicting the incidence rather than just the prevalence of cardiovascular disease, with an accompanying emphasis on determining individual risk profiles. A combination of risk factors, including history, age, gender, diabetes, smoking, blood pressure and cholesterol have been identified as absolute risk factors [6]. Secondly, there is evidence from intervention studies that reducing factors such as smoking, blood pressure and lipids will reduce the risk of disease and stroke. Using a decision tree approach, risk assessment charts have been developed coupled with guidelines to enable clinicians to predict risk for their patients [6], with the estimate of risk usually covering a five year period. These charts can then be linked to a computerised decision support system, and to Internet based tools designed for clinicians and patients. From a clinical point of view, it is possible to establish the likely treatment or intervention benefit to be expected on the basis of intervention. Such information can be tailored and personalised, and may serve as a direct motivator for behavioural change in patients. Given the relative progress towards prevention made in CVD, and the lack of progress in mental health field, there is clear need to extend the CVD approach to risk estimation and reduction to the area of depression.

While the decision tree methodology has been widely used to identify modifiable risk factors for CVD, the approach has been rarely used in the mental health domain. There have, however, been attempts to use decision tree methods to predict suicide attempts [7], levels of neuroticism [8], quality of life [9], and late-life depression [4,5]. Decision trees are a family of analytic techniques, which include CHAID (Chi-square Automatic Interaction Detector) and CART (Classification and Regression Trees). They provide estimates of risk by partitioning the sample on the basis of the best predictors of the outcome.

Using a large prospective narrow age cohort study, the present paper has three aims: to establish which of many candidate risk factors predict the continuation or emergence of depression at a four year interval; to determine individual risk profiles based on combination of modifiable and non modifiable risk indicators, and, given that risk factors vary across the lifespan [10], to determine risk profiles across different age groups. To develop the risk model, a range of risk indicators were identified which have individually been found either to predict depression at follow-up or to be associated with the prevalence of depression in community studies. Relevant risk factors shown in cohort studies to predict depression included in this cohort study were initial depression levels [4,11], use of alcohol [11,12], cannabis use [13-15], smoking [16,17], life events [4,18,19], chronic illness [4], medical illness [4,20], low level of education or low levels of mastery [21], employment status or financial pressure [22,23], religious service attendance [24,25], living alone [5], age and gender [10]. Evidence from intervention trials also point to the importance of physical activity in the treatment of depression [26]. Additional health measures such as body mass index have also been implicated as risk factors for depression [27].

## Methods

### Participants

The PATH Through Life Project is a community survey examining the health and well-being of people who are 20-24, 40-44, and 60-64 years of age [28]. Each cohort is being followed up every four years over a total period of 20 years. Participants were sampled from the electoral rolls for the city of Canberra, Australia, and in the neighbouring town of Queanbeyan. Registration on the electoral roll is compulsory for Australian citizens. Results presented here concern the first two waves of interviews, conducted in 1999-2002 and 2003-2006 (recruitment was staggered by age group). At baseline, interviews were completed with 7,485 participants: 2,404 in the 20-24 group, 2,530 in the 40-44 group and 2,551 in the 20-24 group. Participation rates of those who were found to be in the appropriate age ranges were 58.6% for the 20-24 s, 64.6% for the 40-44 s and 58.3% for the 60-64 s.

Wave 2 interviews were completed four years later by 6,715 participants (89.7% follow-up rate): 2,139 (89.0%) 20-24 s, 2,354 (93.0%) 40-44 s and 2,222 (87.8%) 60-64 s. Participants missing the depression measurement at Wave 2 (n = 76, 1.1%), missing the baseline Goldberg depression score (n = 32, 0.5%) or missing education status (n = 2, < 0.1%) were excluded from the analysis, leaving a sample of 6,605. The sample included 2,105 (31.9%) participants in their 20 s, 2,323 (35.2%) in their 40 s and 2,177 (33.0%) in their 60 s, including 3,383 (51.2%) females overall.

### Procedure

Participants were interviewed at a convenient location, usually the participant's home or the Centre for Mental Health Research at the Australian National University. Most of the interview was self-completed on a palmtop or laptop computer. However, testing by the interviewer was required for the physical tests, some of the cognitive tests and a cheek swab used for genetic testing. Approval for the research was obtained from The Australian National University's Human Research Ethics Committee.

### Measures

The outcome measure was presence or absence of major depressive disorder (MDD) at the four-year follow-up. The assessment of MDD was made using the Patient Health Questionnaire (PHQ), a measure that has 73% sensitivity and 93% specificity in detecting MDD [29].

Baseline modifiable risk indicators included: depressive symptoms, tobacco use, alcohol use, marijuana use, Body Mass Index, hypertension and physical activity. Depressive symptoms were assessed using the Goldberg Depression Scale [30], which was categorized into four groups for the analysis (0-1, 2-3, 4-6 and 7-9 symptoms). Based on their response to the item, "Do you currently smoke?", participants were categorized as current smokers or not. A cut-off of eight points on the World Health Organization's Alcohol Use Disorders Identification Test (AUDIT) [31] was used to identify those participants who exhibited harmful or hazardous levels of alcohol consumption. Marijuana use in the past year was identified using a single item, "Have you used marijuana in the past 12 months?" Participants were classified as being overweight if their body mass index (BMI) exceeded 25. Current hypertension was based on both blood pressure measurements and a self-reported item, "Are you currently taking any tablets for high blood pressure?" Low threshold criteria were used to define hypertension, with cut-offs of systolic blood pressure ≥140 mmHg or diastolic blood pressure ≥90 mmHg. Physical activity level was assessed by asking participants how many hours they spent in an average week engaged in mildly energetic, moderate energetic and vigorous physical activity, with examples provided for each level. Responses were categorized in two ways for each level: zero vs. any weekly hours and <3 vs. ≥3 weekly hours.

Background risk indicators measure at baseline included: gender, age group, education, employment status, financial pressure, religious service attendance, self-rated health and life events. Age group consisted of the three age cohorts recruited to the study (20 s, 40 s and 60 s). Years of education was classified into "less than high school" (< 13 years), "high school" (13-<15 years) and "greater than high school" (≥15 years) based on responses to four questions regarding past and current educational attainment. Employment status was categorized in the survey as "Employed full-time", "Employed part-time, looking for full-time work", "Employed part-time", "Unemployed, looking for work", or "Not in the labour force". The part-time employment categories were combined and the not employed categories were combined, resulting in three employment categories: full-time (FT), part-time (PT) and not in the labour force (NILF). Participants were classified as being under financial pressure if they responded "Yes, often" or "Yes, sometimes" to the item, "Have you or your family had to go without things you really needed in the last year because you were short of money?" Participants who attended religious services "once a month", "more than once a month", "once a week" or "more than once a week" were classified as religious attendees. General health status was self-rated using a five-category item, with responses combined into two categories: "excellent"/"very good"/"good" and "fair"/"poor". Stressful life events in the six months prior to the survey were assessed using a list of 16 events, from which categories of "fewer than two events" and "two or more events" were distinguished.

### Analysis

Sample characteristics were tabulated, broken down by presence or absence of major depressive disorder after four years. The decision tree was constructed using the *treedisc *macro in SAS v9.1.3. The *treedisc *macro chooses each of the branches on the basis of the risk indicator with the minimum p-value from the chi-square statistic of that division. Branching stops when there are no risk indicators with a p-value less than 0.1 for division. The minimum sample size for each leaf (node) was specified as n = 50, and branching was limited to five levels. To examine the effectiveness of the decision tree in predicting depression risk relative to conventional methods, the method was compared to a logistic regression that used identical risk indicators. Receiver operating characteristic (ROC) curves for the decision tree and the logistic regression were plotted to assess the performance of each approach with the areas under the curve compared using the method of DeLong, DeLong and Clarke-Pearson [32].

## Results

Sample characteristics are presented in Table 1, showing the prevalence of major depressive disorder broken down by each of the risk indicators. The table shows that the risk of depression after four years was significantly higher for participants who were younger, smoked, used alcohol at a harmful or hazardous level, used marijuana, did not participate in moderate physical activity, rated their health more poorly, had less education, were in less secure employment or under financial pressure, or had experienced more life events.

**Table 1:** Descriptive statistics based on absence or presence of major depressive disorder at the four year follow-up of the PATH cohort

	No major depressive disorder n = 6334		Major depressive disorder n = 271		Chi-square/*F value*	p value
Goldberg depression: M (SD)	2.17	(2.18)	5.08	(2.41)	*461.8*	* < 0.001*
						
Gender						
*Male*	3097	(96.1%)	125	(3.9%)	0.8	0.372
*Female*	3237	(95.7%)	146	(4.3%)		
Age group						
*20-24*	1978	(94.0%)	127	(6.0%)	46.6	< 0.001
*40-44*	2221	(95.6%)	102	(4.4%)		
*60-64*	2135	(98.1%)	42	(1.9%)		
Current smoker						
*Yes*	1167	(92.3%)	98	(7.7%)	52.8	< 0.001
*No*	5167	(96.8%)	173	(3.2%)		
Harmful/hazardous alcohol use						
*Yes*	1182	(92.7%)	93	(7.3%)	40.7	< 0.001
*No*	5144	(96.7%)	178	(3.3%)		
Marijuana user						
*Yes*	818	(92.3%)	68	(7.7%)	33.2	< 0.001
*No*	5516	(96.5%)	203	(3.5%)		
Do mild physical activity						
*Yes*	3438	(96.4%)	130	(3.6%)	4.2	0.041
*No*	2743	(95.3%)	134	(4.7%)		
Do moderate physical activity						
*Yes*	3700	(96.5%)	134	(3.5%)	8.5	0.004
*No*	2201	(95.0%)	116	(5.0%)		
Do vigorous physical activity						
*Yes*	2968	(96.1%)	122	(3.9%)	0.4	0.518
*No*	3207	(95.7%)	143	(4.3%)		
3+ hours moderate physical activity						
*Yes*	1940	(96.1%)	78	(3.9%)	0.3	0.580
*No*	3961	(95.8%)	172	(4.2%)		
Subjective health rating						
*Excellent*	1303	(98.9%)	14	(1.1%)	183.6	< 0.001
*Very good*	2658	(97.2%)	76	(2.8%)		
*Good*	1820	(94.8%)	99	(5.2%)		
*Fair*	479	(88.1%)	65	(11.9%)		
*Poor*	73	(81.1%)	17	(18.9%)		
Hypertension						
*Yes*	2442	(96.2%)	96	(3.8%)	0.3	0.587
*No*	3525	(95.9%)	149	(4.1%)		
Overweight (BMI>25)						
*Yes*	2839	(96.2%)	112	(3.8%)	1.8	0.179
*No*	3049	(95.5%)	143	(4.5%)		
Education status						
*Less than high school*	1165	(94.6%)	66	(5.4%)	30.9	< 0.001
*High school graduate*	2455	(94.8%)	135	(5.2%)		
*University graduate*	2714	(97.5%)	70	(2.5%)		
Employment status						
*Full-time*	3212	(96.5%)	115	(3.5%)	9.3	0.010
*Part-time*	1436	(95.8%)	63	(4.2%)		
*Not in the work force*	1686	(94.8%)	93	(5.2%)		
Financial pressure						
*Yes*	1184	(91.7%)	107	(8.3%)	71.4	< 0.001
*No*	5150	(96.9%)	164	(3.1%)		
Life events						
*0*	2452	(98.1%)	48	(1.9%)	85.6	< 0.001
*1*	1768	(96.7%)	61	(3.3%)		
*2 or more*	2114	(92.9%)	162	(7.1%)		
Religious service attendee						
*Yes*	1249	(96.4%)	47	(3.6%)	0.9	0.335
*No*	5085	(95.8%)	224	(4.2%)		

As expected, those who were depressed after four years had initial depression symptom scores more than twice as high as those who were not depressed.

**Figure 1:**
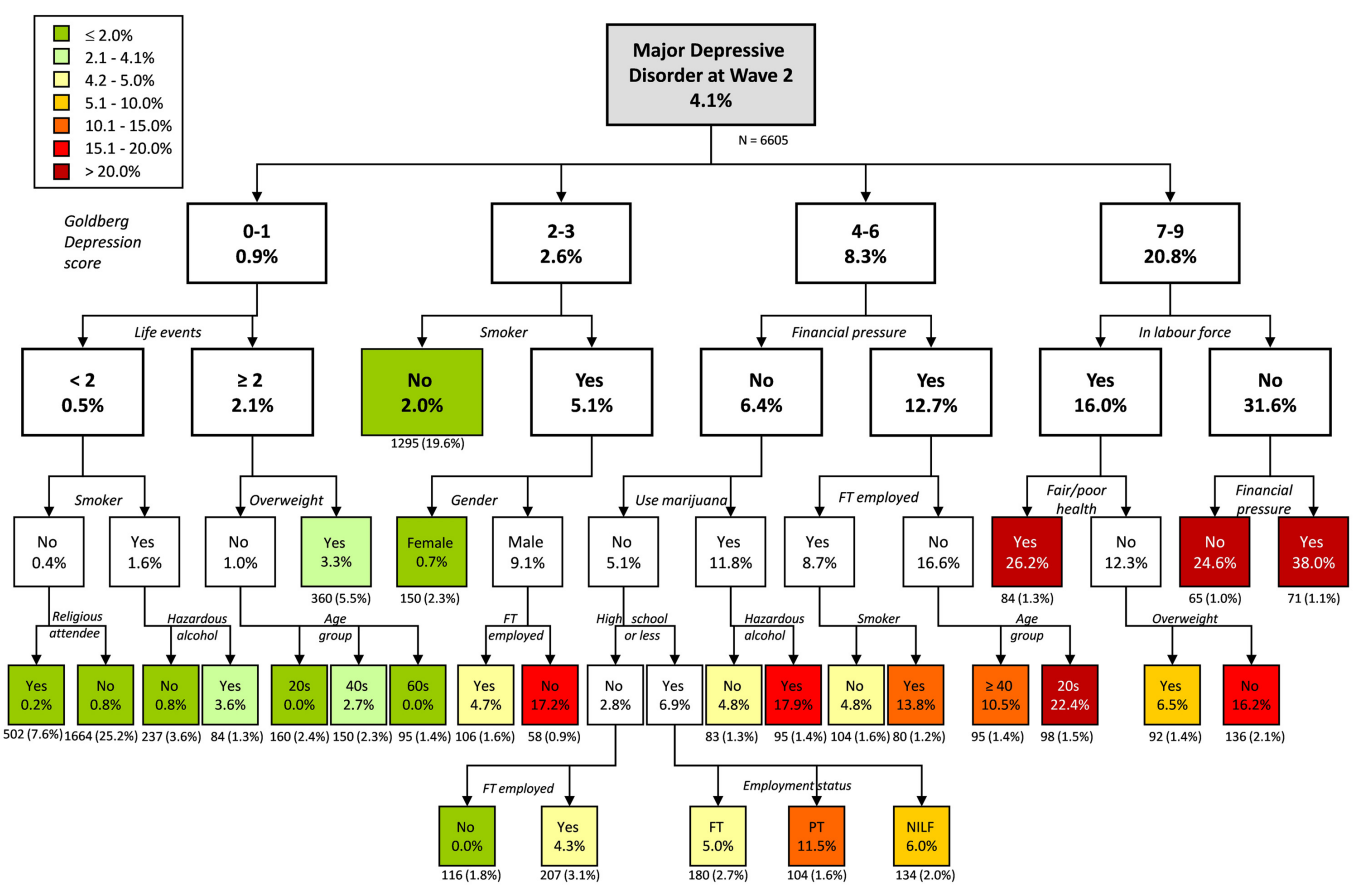
**Decision tree predicting the risk of major depressive disorder at the four year follow-up of the PATH cohort**.

The decision tree resulting from the *treedisc *analysis is shown in Figure 1. Initial depression symptoms were most strongly associated with risk of depression. However, within symptom categories there was a large range of risk profiles. For example, male smokers who were not full-time employed and had only 2-3 symptoms were at a 17% risk of having major depressive disorder after four years. This risk was less than 5% for those engaged in full-time employment. Likewise, participants with 4-6 symptoms who were under financial pressure and used marijuana had an 18% risk of depression if they were using alcohol to a harmful/hazardous extent, while the risk was less than 5% for those not using harmful/hazardous amounts of alcohol. While those with 7-9 symptoms had a 21% risk overall of having depression after four years, the risk is as low as 7% for certain subgroups, such as those who are in good physical health and employed.

Significantly, factors associated with depression risk were different depending on the initial level of symptoms. Substance use--particularly smoking and alcohol use--appear as predictors in all but the highest symptom level group. Employment status, financial pressure and education also feature prominently, particularly for those with higher symptom levels. Life events, religious service attendance, age group, weight and self-rated health also appear as predictors in the tree. However, physical activity and hypertension did not distinguish between depression risk groups and were omitted from the tree.

In order to examine the performance of the decision tree approach in predicting depression risk, it was compared to a conventional logistic regression model limited to additive effects of each variable. The regression included risk indicators that appeared once or more in the decision tree, that is, Goldberg Depression Scale category, smoking status, marijuana use, harmful/hazardous alcohol use, age group, gender, employment status, financial pressure, education level, life events, overweight, self-rated health and religious service attendance. In the logistic regression, Goldberg Depression symptom category (OR_0-1 vs. 2-3 _= 2.8,  = 9.1, *p *= .0025; OR_0-1 vs. 4-6 _= 7.8,  = 27.2, *p *< .0001; OR_0-1 vs. 7-9 _= 16.3,  = 97.5, *p *< .0001), harmful/hazardous alcohol use (OR = 1.6,  = 6.8, *p *= .0090), age group (OR_20 s vs. 60 s _= 2.3,  = 4.3, *p *= .0378; OR_40 s vs. 60 s _= 2.4,  = 7.7, *p *= .0056), full-time employment (OR_FTvs.NILF _= 2.1,  = 8.3, *p *= .0040), financial pressure (OR = 1.4,  = 4.4, *p *= .0355), and poor/fair self-rated health (OR = 1.9,  = 14.3, *p *= .0002) were significantly associated with major depressive disorder after four years.

**Figure 2:**
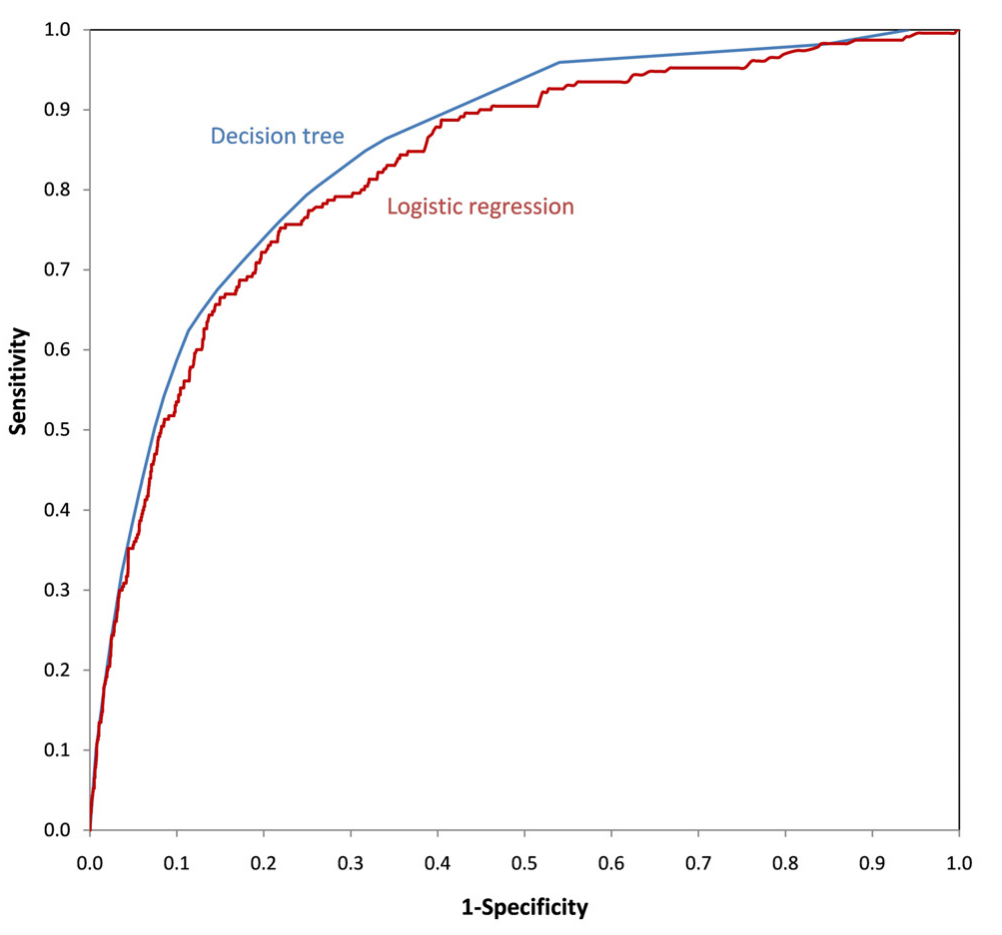
**Receiver Operating Characteristic curves for the decision tree and the logistic regression model**.

Figure 2 shows the ROC curves for the logistic regression and the decision tree. The standard against which sensitivity and specificity were calculated for both curves was major depressive disorder at wave 2 as diagnosed by the PHQ. From the logistic regression, predicted probabilities were output and used to create the curve. For the decision tree, the risk at the endpoints of the tree (shaded leaves in Figure 1) were used as the predicted probabilities for each individual in that leaf. The areas under the curves were 0.850 for the decision tree and 0.828 for the logistic regression. The area under the decision tree ROC curve was significantly greater than the area under the logistic regression ROC curve ( = 7.5, *p *= .006).

## Discussion

Decision tree methodology successfully categorized participants in the PATH cohort into a wide range of depression risk groups, distinguishing subgroups of participants with virtually no risk through to groups with almost 40% risk of having major depressive disorder four years after their status on a raft of risk indicators was ascertained. Both background and potentially modifiable risk indicators were used to form categories. The importance of individual risk indicators in predicting status at wave 2 was dependent on previous level of symptoms. The decision tree showed a modest overall performance but a usable advantage in cut regions having clinical or preventive utility. Furthermore, risk factors that may have been overlooked by a logistic regression, such as gender, smoking status and education status, were important predictors of risk for certain subgroups of participants. While adding higher-order interaction terms to the logistic regression model may bring it closer to the decision tree model, choosing which interactions to include is problematic, requiring a selection strategy and leading to a decrease in parsimony. The decision tree model provides a way to identify important interactions and breaks down risk profiles into manageable categories with high clinical utility. This method has been very effective for identifying CVD risk and now shows promise in identifying mental health risk. This paper further contributes by its focus on three lifespan groups, its emphasis on determining the effects of both modifiable risk factors and non-modifiable risk factors, and its aim to develop a tool to assist patients and their clinicians to determine absolute risk. Unlike previous models of depression risk that studied only those with late-life depression [4,5] this model is applicable across a broad adult age range.

The present findings are consistent with the previous studies examining the determinants of depression risk in older populations. Schoevers et al. [4] found that initial depression symptoms most strongly distinguished depression risk, with illness and disability, living situation and female gender also having an impact. Smits et al. [5] found that anxiety symptoms, functional impairment, chronic illness, low mastery, low education and having no partner were the risk factors that best predicted depression risk. These studies echo the finding of initial symptoms being most strongly associated with the risk of depression. However, among these elderly cohorts, health status and living situation had a larger impact on depression risk than was found in the present study. Substance use, employment and life pressures were not examined in the two studies of late-life depression, yet these factors contributed strongly to predicting depression risk in the present study.

The most highly predictive risk factor for future depression was the initial symptom score severity. While it may appear circular to include participants with subclinical or incident depression in the analysis, the modifiability of depression symptoms through treatment is a vital way to decrease the prevalence of major depressive episodes. The findings support the need for increased access to treatment through interventions that provide targeted prevention programs and increased mental health literacy. Furthermore, while sub-clinical symptom levels are a powerful predictor of developing future caseness, the present study indicates that there are subgroups with low symptom levels that still have a markedly increased risk of experiencing a future major depressive episode and subgroups with high symptoms levels with low risk of depression. Although a baseline measure of depression caseness was not available for this cohort, future research could examine whether there are differences in the predictors of new versus existing cases of depression.

There are several limitations in applying the decision tree method to treatment and prevention programs. Most importantly, the causal relationships between depression and risk behaviours, such as substance use, employment status and physical health, may be bidirectional to some extent. This limitation is mitigated by the longitudinal nature of the present data, in that the depression outcome was assessed four years after the initial measurements were taken. Nevertheless, care must be taken in stating the effects of making lifestyle or behavioural changes, such as quitting smoking, reducing alcohol intake or finding full-time employment. The outcome measure poses additional methodological limitations, specifically, a full clinical interview could not be used due to resource limitations, and depressive episodes that occurred within the four years between measurement occasions may not have been captured. These missed episodes may have led to an underestimation of absolute risk. Further validation of the model in other cohorts or specific populations will enhance the applicability of using the model to predict risk. Finally, this analysis was confined to a restricted set of modifiable risk indicators for depression and variables which might delimit sub-groups with differential risk profiles. There may be additional variables that would improve the predictive accuracy of the model, including psychological indicators such as personality, ruminative style and mastery, however the present analysis was intended to focus on factors that are more amenable to modification.

## Conclusion

The decision tree method was useful in assessing the risk of major depressive disorder over four years. This method has potential to be developed into a predictive tool for use by both clinicians and patients. Such a tool would have high clinical utility by providing customized feedback to mental health consumers which focuses on personal attributes which put them at risk and identifies possible ways in which they might modify aspects of their lifestyle to reduce their risk. It would highlight to clinicians the importance of different combinations of characteristics and the different roles of risk indicators for individuals in different circumstances. Prevention or early intervention programs may also be tailored based on the assessed level of risk by focussing on the specific modifiable factors that are driving that risk. Although predicting depression risk appears to be more complex and multifaceted than predicting CVD risk, the decision tree methodology used for CVD risk assessment provides a useful framework for depression screening. While further validation is required in other samples, there is much promise in developing these models to guide future prevention and treatment efforts aimed at reducing the prevalence of depression.

## Competing interests

The authors declare that they have no competing interests.

## Authors' contributions

PJB drafted the manuscript and performed the analysis; HC revised the manuscript and contributed to the design of the study; AJM contributed to the design of the study, contributed to the analysis and revised the manuscript. All authors read and approved the final manuscript.

## Pre-publication history

The pre-publication history for this paper can be accessed here:

http://www.biomedcentral.com/1471-244X/9/75/prepub
